# The Physiological Mechanisms and Hurdles of Efficient Water–Nitrogen Utilization in Maize Production: A Review

**DOI:** 10.3390/plants14131899

**Published:** 2025-06-20

**Authors:** Xichao Sun, Qian Zhao, Jia Gao, Zheng Liu

**Affiliations:** 1 Agro-Environmental Protection Institute, Ministry of Agriculture and Rural Affairs, Tianjin 300191, China; sunxichao@caas.cn (X.S.); zhaoqian01@caas.cn (Q.Z.); 2 State Key Laboratory of Efficient Utilization of Agricultural Water Resources, China Agricultural University, Beijing 100083, China; gaoj0803@163.com; 3 National Field Scientific Observation and Research Station on Efficient Water Use of Oasis Agriculture in Wuwei of Gansu Province, Wuwei 733009, China; 4 Institute of Crop Sciences, Chinese Academy of Agricultural Sciences, Beijing 100081, China; 5 Key Laboratory of Crop Physiology and Ecology, Ministry of Agriculture and Rural Affairs, Beijing 100081, China

**Keywords:** maize, nitrogen, deficit irrigation, ear development, floret primordium, signal transduction, cytokinin

## Abstract

Maize (*Zea mays* L.) is one of the most important staple food crops globally. One-third of global maize production is located in areas with high or extreme water scarcity and concurrently faces the challenge of low nitrogen use efficiency. Therefore, achieving synergistically high-efficiency water and nitrogen utilization in maize production is of great significance for agricultural sustainable development and global food security. In recent years, more articles have focused on the physiological mechanisms and management practices of efficient water and nitrogen utilization in maize. Unfortunately, there is a relative scarcity of research on the interactive effects between water and nitrogen on the development of young ears, which plays a crucial role in maize productivity. By compiling the most pertinent publications, this review endeavors to consolidate the existing knowledge on the interactive effects between water and nitrogen on maize production. Moreover, it advances potential physiological mechanisms and strategies for high-efficiency water and nitrogen utilization in terms of root system functioning, phytohormones, metabolism, and organ development. The changes in the availability of water and nitrogen have a significant impact on the development of young ears during the critical period, which in turn directly determines the grain number per ear and grain weight.

## 1. Introduction

Maize (*Zea mays* L.) plays a pivotal role in ensuring global food security, economic stability, and industrial development. As one of the most important cereals, maize provides over 30% of dietary calories for 4.5 billion people across 94 developing countries, serving as a primary staple for 900 million impoverished consumers [1]. Over 60% of global maize production is allocated to animal feed, linking crop cultivation to livestock and poultry industries, while its industrial applications, such as bioethanol, bioplastics, and pharmaceuticals, suggest strategic importance in modern economies [2]. However, rising demand driven by population growth, dietary shifts toward animal protein, and biofuel mandates has strained global maize supplies, with prices surging by 43% since 2008, exacerbating food insecurity in low-income regions [2]. In China, maize has emerged as the largest grain crop, contributing over 40% of national grain output and sustaining critical value chains in feed, bioenergy, and manufacturing [3]. Between 2019 and 2024, China’s maize production grew at an annual rate of 2.5%, reaching 294.92 million tons, driven by genetic improvement, optimized planting patterns, and policy incentives, including producer subsidies and high-standard farmland construction [4]. Domestic demand, particularly feed and industrial uses, continues to outpace supply, necessitating imports that reached 13.64 million tons in 2024, primarily from Brazil and Ukraine. Moreover, resource constraints, such as groundwater depletion and climate-induced yield variability, threaten long-term agricultural sustainability [5]. China’s maize sector exemplifies the dual mandate of securing food self-sufficiency while navigating global market dynamics. The government’s emphasis on “sustainable intensification” aligns with the Global Sustainable Development Goals (SDGs), promoting water-efficient irrigation, precision agriculture, and circular bioeconomy models to reconcile productivity with environmental stewardship [6].

Water scarcity has emerged as a critical constraint to global maize production, threatening food security and agricultural sustainability. Under climate change, the frequency and intensity of extreme weather events, including prolonged droughts and erratic rainfall patterns, have significantly increased, exacerbating water resource limitations in key maize-growing regions [7]. Maize is particularly vulnerable to water deficits during critical growth stages, such as flowering and grain filling, leading to yield losses of 10–15% under moderate drought conditions [8]. In China, for instance, the 2022 extreme drought, which was the most severe since 1961, highlighted the fragility of maize production systems despite advanced irrigation interventions [9]. Similarly, in sub-Saharan Africa, where smallholder farmers dominate agriculture, water scarcity and inadequate irrigation infrastructure have perpetuated low maize yields, further intensifying food insecurity [2]. To address these challenges, deficit irrigation (DI), that is, a water-saving strategy that applies controlled water stress during drought-tolerant growth stages, has gained prominence as a means to balance water conservation with yield stability. Research has demonstrated that DI can enhance water use efficiency (WUE) by optimizing crop physiological responses to water scarcity. For example, previous studies document that DI improved maize WUE by 7.43%, albeit with an 8.74% yield reduction compared to full irrigation in China [5,10]. However, the efficacy of DI depends on strategic timing and the severity of water stress. A 75% crop evapotranspiration (ETc) substitution level with a 30 mm irrigation threshold minimizes yield losses (≤10%) while achieving 25% water savings and 12% higher irrigation water productivity, particularly in regions with annual precipitation of 400–800 mm and loamy soils. These findings highlight the importance of modulating DI strategies to local climatic and edaphic conditions. Despite these advancements, challenges persist in scaling DI and related technologies. For instance, water-saving irrigation systems often face the “efficiency paradox”, where reduced field-level water consumption is offset by increased return flows or expanded irrigation areas, limiting net water savings at regional scales [10]. Additionally, socioeconomic barriers, such as limited access to financing and mechanization in developing regions, hinder the adoption of advanced irrigation strategies. Global initiatives emphasize the urgency of integrating climate-smart irrigation technologies, policy frameworks, and cross-sectoral collaboration to build resilient agri-food systems [11].

Nitrogen (N) is a critical macronutrient for maize growth, directly influencing photosynthetic efficiency, biomass accumulation, and grain yield. Globally, maize production relies heavily on N fertilizers, with average application rates exceeding 300 kg ha^−1^ in regions like the North China Plain [12]. However, only 30–50% of applied N is utilized by crops, with the remainder lost via leaching, volatilization, or denitrification. Traditional practices, such as split applications of urea, improve N availability but still exhibit inefficiencies due to mismatched timing between nutrient release and crop demand. Excessive or inefficient N application has led to environmental degradation and reduced N use efficiency (NUE), necessitating a deeper understanding of its physiological mechanisms and management strategies. In maize, N deficiency reduces leaf chlorophyll content, impairs stomatal conductance, and lowers the net photosynthetic rate, thereby limiting radiation use efficiency (RUE) and WUE [5,13]. Reduced N availability decreases the activity of N metabolism enzymes, such as glutamine synthetase, which disrupts N assimilation and delays leaf senescence during the grain-filling stage. Moreover, N governs root architecture. The optimal N supply promotes deeper root distribution, enhancing nutrient and water uptake, particularly in semi-arid regions. Conversely, excessive N application disrupts root–soil interactions, leading to shallow root systems and increased susceptibility to environmental stress. Advancements in agronomic practices and maize breeding have targeted NUE improvement. Meanwhile, root-zone N management strategies, which adjust fertilizer rates based on real-time soil mineral N levels, achieved NUE values of 66%, which is twice that of conventional practices, while maintaining yields of 12.6–13.5 Mg ha^−1^ [14,15]. At the genetic level, the discovery of the THP9 gene from teosinte, that is, the wild maize ancestor, has opened new avenues for breeding high-NUE cultivars. THP9 encodes asparagine synthetase 4 (ASN4), a key enzyme in N metabolism. Field trials showed that hybrids expressing the THP9-T allele exhibited more than 20% higher grain protein content under low-N conditions, offering a genetic solution to reconcile high yield with reduced N fertilizer dependency [16].

Enhancing both WUE and NUE in maize production is critical for addressing global challenges such as water scarcity, environmental pollution, and sustainable food security. In semi-arid and arid regions, water and N are the primary limiting factors for crop productivity. In regions like Northern China, average maize WUE and NUE remain suboptimal at 1.83 kg·m^−3^ and 55.66 kg·kg^−1^, respectively, indicating significant potential for improvement [10,12]. Coordinated management of water and N resources is essential to balance productivity, resource conservation, and ecological resilience, particularly under climate change scenarios characterized by erratic rainfall and rising temperatures [2,5,11]. For instance, ridge-furrow film mulching combined with biochar amendment enhances soil water storage in surface layers, promotes root water uptake, and reduces N losses by 20.6–46.9%, thereby boosting WUE and NUE simultaneously [17]. In addition, deficit irrigation (e.g., 0.8 evapotranspiration) paired with moderate N rates (180 kg ha^−1^) and biochar application increases WUE by 11–15% and N recovery efficiency by 56.8–63.1% without compromising yield. One of the potential mechanisms is that changes in the availability of water and N in the root zone induce hydraulic signaling, stimulating root proliferation and hormonal responses, thereby enhancing drought tolerance and N assimilation [18]. Synergistic improvements in WUE and NUE not only optimize resource utilization but also mitigate trade-offs between yield and environmental sustainability. Despite progress, challenges still persist in scaling site-specific strategies across diverse agroecological zones. Optimizing N use in maize hinges on understanding its physiological roles and adopting appropriate N management, especially considering the interaction between N and water. Integrating deficit irrigation with N management strategies may offer a holistic approach to achieving sustainable maize production. Future research must address knowledge gaps, including the interplay between N availability and water stress, plant growth and development, resource use efficiency under DI, and ecological and economic budgets in technology dissemination. This review synthesizes current research on integrated strategies to enhance WUE and NUE in maize systems, explores underlying physiological and ecological mechanisms, and identifies gaps in knowledge to guide future research.

## 2. Grain Number Drives the Response of Maize Productivity to Water and Nitrogen Stresses

The increments in grain number (GN) have played a more dominant role than the grain weight (GW) in driving maize productivity over the past several decades [19]. This trend is particularly evident in hybrid maize, where selection for prolificacy and stress tolerance has prioritized increases in grain set over individual grain size. In terms of maize hybrids released from 1946 to 2015, the modern genotypes exhibit prolonged exponential growth phases of ear development, enabling greater partitioning of assimilates to grain initiation rather than grain filling. This implies that GN is more responsive to genetic manipulation, as it is determined during the early critical period when source–sink relationships and hormonal regulation are highly plastic. The critical period is the four weeks bracketing the silking stage of maize. During this period, the growth and development of young ears determine the final number and weight of grains [19]. The superior contribution of GN to the stability of maize grain yield is further underscored by its heightened sensitivity to environmental fluctuations, particularly under varying water and N. In addition, GN exhibits greater phenotypic plasticity than GW. In semi-arid regions, optimizing N forms, expressed in the ratio of nitrate to ammonium, significantly enhances GN by 8.3–36.1%, whereas GW improvements are marginal [20,21]. GN accounted for 60–80% of yield variation under water-limited conditions, compared to GW’s 20–40% contribution. This differential response is mechanistically linked to GN’s reliance on early-stage resource acquisition and hormonal signaling (e.g., auxin and cytokinin), which are highly susceptible to abiotic stressors. N availability further amplifies the primacy of GN in yield determination. Studies on N-stressed hybrids demonstrated that GN reductions under low N exceeded 40%, whereas GW declined by only 10–15%. This aligns with findings that GN strongly correlates with pre-silking N uptake, while GW depends on post-silking N remobilization [19].

### 2.1. Grain Set During the Critical Period

**Figure 1:**
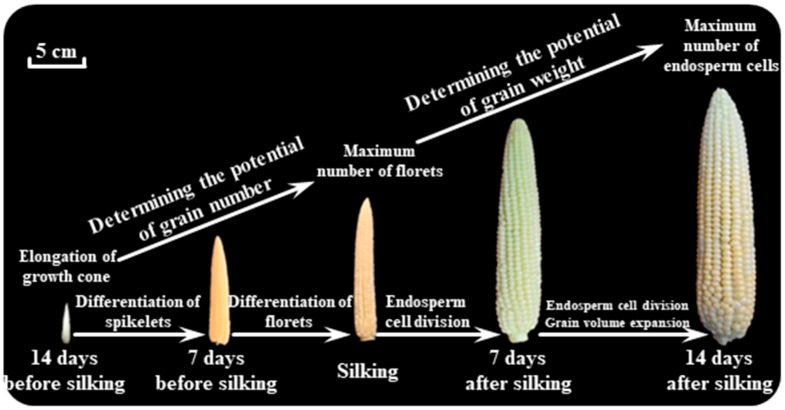
Maize young ear differentiation during the critical period and its potential impact on grain number and grain weight (modified from Liu et al., 2021 [30] and 2022 [31]).

Grain set during the critical period of maize development, spanning from ear differentiation to silking, is a pivotal determinant of final yield. This phase is characterized by the formation of floret primordia, synchronization of reproductive organ development, and the establishment of sink capacity for grain filling (Figure 1). Over the past decade, significant progress has been made in elucidating the physiological and molecular mechanisms underlying the regulation of grain set, particularly focusing on plant growth dynamics, N accumulation and remobilization, ear development, and environmental interactions. The critical period is marked by rapid vegetative growth and reproductive organ initiation. Our previous studies demonstrate that N availability during this phase profoundly influences ear development and grain set. Moreover, insufficient N supply at the critical period reduces floret primordia formation and increases grain abortion due to impaired carbohydrate and N allocation to the ear [22,23]. Low-N-tolerant cultivars like Zhengdan958 exhibit superior N remobilization efficiency from vegetative tissues to the developing ear compared to less tolerant varieties, ensuring adequate N supply for floret differentiation during the critical period. These findings underscore the importance of synchronizing N uptake and allocation with ear developmental demands. Ear growth during the critical period is tightly regulated by hormonal signaling, particularly auxin and cytokinin (CK). Recent breakthroughs in gene cloning have identified *YIGE1* and *YIGE2*, homologous genes that redundantly modulate ear length and the grain row number by influencing auxin homeostasis in the ear meristem [24]. Knockout of both genes via CRISPR-Cas9 significantly reduces auxin and its derivatives, including indole-3-acetic acid (IAA)-Asp and OxIAA in young ears, leading to fewer florets and shorter ears. This auxin-mediated regulation aligns with earlier observations that drought stress disrupts auxin-related gene expression, such as *SAUR* and *Expansins*, delaying silk elongation and exacerbating the anthesis-silking interval (ASI) [25]. Such hormonal crosstalk highlights the dual role of auxin in coordinating ear morphogenesis and stress resilience. In the early stages of ear primordium formation, a sufficient level of CK ensures that the cells in the meristematic region divide actively, laying the foundation for the proper development of the ear structure [19]. Moreover, CK is involved in regulating the differentiation of different tissues within the female ear. It can influence the transition of meristematic cells into specific cell types, such as those of the ovule and the surrounding tissues. A proper balance of CK is required for the normal differentiation of florets into fertile structures. Environmental factors, such as water, temperature, and solar radiation, interact with genetic pathways to modulate grain set. When the temperature is higher than 36 °C during the critical period, developmental transitions accelerate, but the grain set of maize is reduced by 10–23% [26,27]. Genetic studies on glutaredoxins, such as *ZmGRX2* and *ZmGRX5*, further reveal redox-dependent regulation of meristem activity, where oxidative stress disrupts transcriptional networks governing ear differentiation [28]. Additionally, targeted editing of promoter regions in genes like *CLV* has emerged as a novel strategy to fine-tune stem cell activity, balancing ear morphology and yield potential without compromising developmental stability. Similarly, drought and N deficiency also disrupt the CK-mediated regulatory pathways in maize female ear development [29]. Despite progress, gaps remain in understanding the spatiotemporal coordination of N remobilization, hormonal gradients, and genetic pathways during the critical period.

### 2.2. Effect of Water Availability on Grain Number of Maize

Water availability during the critical period is a decisive factor in determining GN and, consequently, grain yield in maize. GN, established primarily during the ear differentiation to the silking phase, is highly sensitive to water deficits, which disrupt physiological processes such as photosynthesis, carbohydrate allocation, and hormonal signaling. Over the past two decades, extensive research has elucidated how water scarcity modulates these processes, ultimately influencing floret fertility, grain abortion, and yield stability. Water scarcity during the critical period reduces photosynthetic capacity, limiting carbohydrate production and disrupting the equilibrium between the source, that is, the leaf, and the sink, that is, the young ear. The moderate to severe soil water deficits suppress photosynthetic rates at both leaf and canopy levels, impairing the synthesis of sucrose, starch, etc. [32,33]. Prolonged drought during ear differentiation reduces soluble sugar content in developing florets by 30–50%, particularly in apical (disadvantaged) grains, exacerbating grain abortion. Under water deficit, maize prioritizes carbohydrate allocation to middle and basal grains, sacrificing apical ones to optimize limited resources [7,25,27]. The source–sink imbalance is further aggravated by accelerated developmental transitions under drought, which shorten the window for floret primordia formation [17,22,25]. In addition, water deficits alter hormonal homeostasis, directly affecting floret development and grain set. Abscisic acid (ABA), a key drought-responsive hormone, accumulates under water stress and inhibits cell division in the ear meristem, reducing floret number [34]. Drought-induced ethylene biosynthesis also accelerates silk senescence, widening the ASI and impairing pollination. Meanwhile, suppressing ethylene synthesis via *ZmACO* gene knockdown mitigates ASI elongation and grain loss, underscoring ethylene’s dual role in stress response and grain abortion [35]. The enzymatic machinery governing starch synthesis in developing grains is highly sensitive to water availability. Drought downregulates the activity of adenosine diphosphate glucose pyrophosphorylase (ADPG-PPase) and soluble starch synthase, further leading to grain abortion [36,37]. The water stress results in a 40–60% reduction in ADPG-PPase activity of maize ear, correlating with a 15–25% decline in GN. These findings emphasize the temporal sensitivity of carbohydrate metabolism to water deficits, particularly during the critical period. The root architecture profoundly influences the water uptake capacity and GN under fluctuating moisture conditions. Deep-rooted maize genotypes are capable of accessing subsoil water reserves and exhibit higher floret fertility and grain set under drought [38]. The increases in root biomass in the 0–40 cm soil layer improve water extraction and dry matter accumulation by 20–30%, thereby stabilizing GN and grain yield of maize. Another study also demonstrates that quantitative trait loci associated with root depth (including *qADR1* and *qADR9*) enhance water foraging and GN in rice under drought [39]. Strategic irrigation scheduling and genotype selection are pivotal for mitigating the negative impacts of water stress on GN. The supplemental irrigation during ear differentiation and silking restores photosynthetic rates by 25–40%, rescuing apical grain development [40]. While significant progress has been made, challenges persist in dissecting the spatiotemporal interplay between water availability, hormonal crosstalk, and genetic networks. The interaction signaling pathways involving ABA, auxin, ethylene, and CK to GN of maize under dynamic drought conditions remain poorly characterized. Future research would prioritize multi-omics approaches to unravel these complexities and develop predictive models for GN under climate variability. Integrating physiological insights with precision agriculture tools, such as molecular markers for grain set and sensor-based irrigation, will be critical for sustaining maize productivity in water-limited environments.

### 2.3. Effect of Nitrogen Availability on Grain Number of Maize

N availability during the critical period governs floret primordia formation and grain abortion. Insufficient N limits carbohydrate and N allocation to the ear, reducing fertilized floret survival. The genotypic differences in N responsiveness are attributed to N remobilization efficiency from vegetative organs to reproductive organs. The N-insensitive genotype maintains higher N accumulation rates during the critical period under low N, whereas N-sensitive genotypes experience sharp declines in the ear N allocation rate, leading to apical grain abortion [30,31]. Furthermore, delayed N allocation under low-N conditions reduces ear biomass and GN, highlighting the importance of synchronized N supply with developmental demands. In terms of genotypic difference, deep-rooted cultivars demonstrate enhanced tolerance to drought and N deficit via gains in lateral root growth rate [41,42]. Under drought conditions, N application increases root active absorption area and root vitality, respectively, by 7.5–11.7% and 40.8–44.8%, thereby stabilizing floret fertility and mitigating grain abortion by 19.8–166.4% [43,44,45,46]. The root architecture-related QTLs (including *qADR1* and *qADR9*) further underscore the potential of genetic engineering to enhance N foraging and grain set in rice under drought and N-deficient environments [39]. Optimizing N delivery through slow-release fertilizers, such as urea-formaldehyde polymers, and microbial inoculants has emerged as a sustainable strategy to enhance GN by aligning with maize N demand peaks during the critical period. Concurrently, microbial consortia, such as *Bacillus velezensis*-clay composites, enhance N retention in alkaline soils. Reducing N inputs while applying microbial inoculants upregulates root N-phosphorus transporters and starch synthesis genes, improving GN through enhanced rhizosphere microbial diversity [47]. N availability orchestrates carbon-N balance in developing grains through transcriptional regulators. The transcription factor PBF1, identified in maize endosperm, binds N-responsive promoters to modulate starch and protein synthesis. Under N deficit, PBF1 shifts carbon flux from zein protein synthesis to carbohydrate accumulation by repressing *sugary1* and starch branching enzyme genes, thereby stabilizing grain development despite N scarcity [48]. This regulatory flexibility ensures grain survival but may compromise protein content, underscoring the trade-off between GN and quality under suboptimal N conditions. Additionally, glutaredoxins (including *ZmGRX2* and *ZmGRX5*) mediate redox-dependent meristem activity, linking N metabolism to oxidative stress resilience during ear differentiation [28]. Despite progress, gaps persist in understanding the spatiotemporal coordination of N signaling; hormonal crosstalk involving ABA, ethylene, and CK; and environmental interactions. Future research should integrate multi-omics approaches to decode these networks, enabling the design of N management strategies and N-smart maize varieties tailored to diverse agroecosystems.

### 2.4. The Potential Mechanism of Regulating Young Ear Differentiation Through Signal Transduction Rather than Nutrition

In addition to serving as a nutrient, N also acts as a signal to regulate the differentiation of ears and florets in maize. During the critical period, the absolute amount of N required for the early development of the young ear is very low, accounting for only 0.02% of the N accumulation within the maize plant. However, slight N deficiency could significantly inhibit the growth and development of young ears, which occurs earlier than a decrease in N concentration in vegetative organs, such as leaves [30,49,50]. This temporal and spatial specificity suggests that N signaling, rather than mere nutritional supply, governs the ear’s developmental plasticity under fluctuating N conditions. Mechanistically, the development of young ears might predict the N availability in the coming period through some form of signal transduction, and then specifically regulate its growth and development, such as accelerating or reducing the growth rate, promoting or inhibiting the differentiation of floret primordia [51,52]. N availability directly influences hormonal homeostasis, particularly CK and auxin dynamics, which are pivotal for floret initiation and survival. For example, N promotes the expression of genes, including *IPT9* and *CKX12*, which increase the concentration of CK in the young ear of maize, thereby increasing GN [53]. When the N availability is low, the contents of aspartic acid, asparagine, glutamine, etc., in the cob and grains are significantly reduced, and the number of floret primordia decreases. Meanwhile, the number of aborted grains increases. Conversely, higher N availability increases the amino acid content of the young ear and promotes the differentiation of floret primordia and grain setting [54,55]. N signaling interacts with the auxin pathways. The *YIGE1* and *YIGE2* genes regulate auxin biosynthesis in the ear meristem and are indirectly modulated by N availability, affecting ear length and grain rows. This interplay underscores the integration of nutrient signaling and hormonal networks in shaping ear architecture. Emerging evidence highlights the role of redox-sensitive proteins in N signaling. Glutaredoxins (*ZmGRX2* and *ZmGRX5*) modulate the redox state of transcription factors like FEA4, which governs meristem activity and floret differentiation [28]. Under optimal N conditions, GRX-mediated reduction in FEA4 enhances its DNA-binding capacity, activating genes critical for ear development. Conversely, N limitation disrupts this redox balance, impairing meristem function and reducing GN. Additionally, the transcription factor PBF1, which coordinates carbon-N partitioning in grains, is regulated by N availability, shifting metabolic flux toward starch synthesis under N scarcity to stabilize ear development and grain formation. The key gaps remain in understanding the spatiotemporal coordination of N signaling and hormonal crosstalk. The recent discovery of *NGR5*, a gene mediating N and gibberellin synergy in rice, offers a model for maize research, suggesting that analogous pathways may enhance N use efficiency without compromising yield [56]. Numerous studies have demonstrated that N promotes the differentiation of floret primordia and the formation of grains in the young ear of maize. The response of the development of the young ear of maize to N availability during the critical period is very rapid, and N directly regulates the early development of the ear as a signal rather than a nutrient. Therefore, the signal transduction and regulatory mechanism of the response of floret differentiation in the young ear of maize to N needs to be further explored.

## 3. Deficit Irrigation Enhances Water Use Efficiency While Maintaining Grain Yield of Maize

### 3.1. Deficit Irrigation Affects Maize Productivity and Water Use Efficiency

Deficit irrigation (DI), a strategy that applies water below crop requirements during drought-tolerant growth stages, has emerged as a promising approach to balance water conservation and yield stability. DI aims to maximize crop water productivity by strategically reducing irrigation during periods of low sensitivity to water stress, thereby conserving resources without significant yield penalties. In semi-arid regions, such as western Nebraska, DI improves irrigation water productivity by 22–47% compared to full irrigation, despite yield reductions of 2–33% depending on climatic conditions [57]. This trade-off underscores the importance of tailoring DI regimes to local environmental and crop physiological thresholds. DI triggers stomatal closure to reduce transpiration, conserving soil moisture at the expense of photosynthetic carbon assimilation. However, genotypes with improved stomatal control can maintain higher photosynthetic rates under moderate stress, mitigating yield losses [58]. The physiological and agronomic responses of maize to DI are complex, involving trade-offs between water conservation and yield stability. The moderate DI reduces water input by 25% while mitigating 10% of yield losses, primarily by optimizing stomatal conductance and photosynthetic efficiency during the critical period [36,40]. Maize exhibits differential sensitivity to DI depending on the growth stage at which water stress is imposed. Reproductive stages are particularly vulnerable, with water deficits during these periods leading to significant grain abortion and reduced GN [22]. However, DI applied during vegetative stages may induce beneficial stress acclimation, improving later drought resilience. The accumulation of osmolytes (including proline and soluble sugars) and ABA signaling helps maintain cell turgor and protect metabolic functions during water stress. These mechanisms are linked to WUE improvements. DI shifts biomass allocation toward reproductive organs, prioritizing young ear development over vegetative growth. This reallocation is modulated by hormonal pathways and resource availability, influencing final GN [59]. DI enhances WUE by reducing non-productive water loss through transpiration while maintaining carbon assimilation. Drip irrigation combined with DI has proven particularly effective, increasing WUE by 15–25% compared to conventional flood irrigation [60,61]. This improvement is attributed to stomatal regulation, root–shoot signaling, and delayed leaf senescence. Integrating DI with complementary practices such as mulching (e.g., rice straw) and foliar treatments (e.g., potassium bicarbonate sprays) further enhances WUE by improving soil moisture retention and osmotic adjustment. The challenges persist in scaling DI practices. Additionally, regional disparities in technological adoption, as observed in Gansu Province, China, where WUE improvements rely heavily on technical innovation, underscore the need for context-specific strategies [62,63].

### 3.2. Deficit Irrigation Affects Architecture and Function of Maize Root System

DI directly influences the architecture and function of the maize root system, which governs water and nutrient acquisition, stress resilience, and overall plant productivity. DI reduces the total root length and seedling root number but increases the root–shoot ratio (R/S), favoring resource allocation to roots during stress [64]. Moderate DI during the seedling or jointing stages in drip-irrigated maize increased R/S by 12–25%, promoting deeper root penetration into subsoil layers to access moisture and nutrients. This vertical redistribution is critical in arid regions where subsoil water reserves buffer against episodic drought. Under DI treatment, maize develops thicker roots with higher branching density in moist soil zones, enhancing hydraulic conductivity and nutrient uptake efficiency [61,65]. These structural adjustments are often accompanied by compensatory root growth upon rewatering, restoring biomass accumulation and metabolic activity. DI stimulates root vigor, which metabolic upregulation enables roots to maintain ion uptake and energy metabolism under water stress. Concurrently, osmotic adjustment via proline and soluble sugar accumulation preserves cell turgor, sustaining root elongation even as the soil moisture declines. ABA plays a pivotal role in DI-induced root remodeling, which suppresses shoot growth while promoting lateral root formation and stomatal closure [66]. In addition, ABA-mediated signaling enhances root hydraulic conductivity by upregulating aquaporin expression, improving water transport efficiency under DI. Fertilization strategies synergize with DI to amplify root functionality. Combined N and phosphorus application under DI enhances the root length, surface area, and activity, enabling deeper exploitation of soil moisture and nutrients. Long-term organic fertilization further improves soil structure and water retention, buffering root systems against DI-induced stress. While DI enhances drought resilience, it imposes trade-offs that require careful management. Excessive or mistimed DI can reduce root biomass by 20–35%, impairing nutrient uptake and young ear development. In saline soils, DI exacerbates osmotic stress by concentrating salts in the root zone, disrupting ion homeostasis and suppressing root elongation [67,68,69]. Moreover, genetic variability in root system architecture plasticity necessitates hybrid-specific DI regimes. Drought-tolerant hybrids exhibit deeper root systems and faster post-stress recovery, whereas shallow-rooted varieties suffer irreversible yield losses under DI. Unresolved questions include the genetic basis of root plasticity under DI and the long-term impacts of DI on soil–root–microbe interactions. Emerging tools like root phenotyping platforms and transcriptomic analyses offer pathways to dissect these mechanisms. DI profoundly reshapes maize root systems, balancing water conservation with functional resilience. By leveraging physiological insights and agronomic innovations, DI could be optimized to secure maize productivity in water-limited futures.

### 3.3. Deficit Irrigation Affects Phytohormone and Metabolism Within Maize Plant

While prior studies have focused on the effects of DI on grain yield, WUE, and root architecture, emerging research highlights its profound influence on phytohormonal dynamics and metabolic reprogramming within maize plants. These biochemical adjustments are critical for stress adaptation, resource allocation, and survival under water-limited conditions. DI induces complex hormonal crosstalk, with ABA emerging as a central regulator of drought responses. Under DI, ABA biosynthesis is upregulated in roots and leaves, triggering stomatal closure to reduce transpirational water loss and redirecting resources to stress tolerance mechanisms [70,71]. Elevated ABA levels correlate with enhanced osmotic adjustment through proline and soluble sugar accumulation, which stabilize cellular structures under water stress [72]. Concurrently, DI suppresses ethylene production by downregulating *S-adenosyl-L-methionine synthetase* (*SAMS*) activity, a key enzyme in ethylene biosynthesis [73]. This suppression may mitigate ethylene-induced senescence, preserving photosynthetic capacity during the critical period under drought. Auxins, particularly IAA, exhibit dual roles under DI. While moderate DI enhances IAA-mediated root elongation to exploit deeper soil moisture, severe water deficits disrupt polar auxin transport, impairing root–shoot communication [71]. CK is downregulated under DI, reducing shoot growth and prioritizing resource allocation to reproductive organs [74]. Such hormonal trade-offs underscore the balance between growth suppression and stress resilience. DI reduces photosynthetic carbon assimilation but enhances sucrose transport to roots, fueling osmotic adjustment and respiratory energy production [75]. Glucose deuterium fractionation in maize reflects altered sucrose-to-starch partitioning under drought, with metabolic flux redirected toward stress-responsive pathways. DI disrupts N uptake but enhances remobilization from vegetative organs to young ears during the critical period. Foliar N application under DI improves N use efficiency by 15–20% by sustaining nitrate reductase activity and amino acid synthesis. However, excessive N exacerbates oxidative stress, necessitating precise fertilization regimes. ABA signaling upregulates proline biosynthesis genes (*P5CS* and *OAT*), while proline stabilizes ABA receptors, creating a feedback loop that amplifies stress tolerance [72]. Ethylene suppression reduces malondialdehyde accumulation, thereby mitigating oxidative damage under DI. Auxin-mediated root proliferation enhances N foraging in deeper soil layers, synergizing with foliar N applications to sustain metabolic activity under DI [76,77]. Exogenous ABA and/or CK application during the critical period enhances drought resilience without yield losses. There are still key knowledge gaps, including the role of jasmonates and brassinosteroids in DI-induced metabolic shifts, and metabolic memory effects where prior DI exposure primes plants for subsequent droughts through epigenetic or hormonal priming. DI profoundly alters maize phytohormone networks and metabolic fluxes, balancing water conservation with physiological resilience. By decoding these mechanisms, researchers can develop targeted strategies to enhance drought adaptation in maize, contributing to sustainable agriculture in water-limited ecosystems.

## 4. Cytokinin May Serve as a Hub for Both Maize Productivity and EfficientWater–Nitrogen Utilization

### 4.1. Dual Role of Cytokinin in Young Ear Development Regulated by Water and Nitrogen Availability

Under varied water and N supply, CK plays a vital role in young ear initiation and development. CK synthesized in roots is translocated to shoots via the xylem, which enhances meristematic activity and promotes the formation of floret primordia. Exogenous CK application increases the number of florets per ear by 20%, likely through the upregulation of *WUSCHEL*-related genes that maintain cell pluripotency in ear meristems [78]. CK also enhances sink strength by stimulating sucrose transport into developing ears, ensuring adequate carbohydrate supply [79,80,81]. Furthermore, CK interacts synergistically with auxins to regulate vascular bundle differentiation. The serious drought stress reduces root-derived CK biosynthesis, leading to lower CK levels in shoots. This decline disrupts the CK-auxin balance, impairing floret primordia differentiation and increasing grain abortion. The maize plants subjected to serious drought during the critical period exhibit a 30–40% reduction in bioactive CKs, including trans-zeatin, in young ears, correlating with a 25% decrease in GN. Similarly, N limitation suppresses CK production by downregulating *ISOPENTENYLTRANSFERASE* (*IPT*) genes in roots, thereby reducing CK transport to shoots. Low CK levels under N stress delay ear development and reduce the number of floret primordia, as observed in field trials, which decreases the ear length by 15% compared with N-sufficient controls [82]. Water stress upregulates *CYTOKININ OXIDASE/DEHYDROGENASE* (*CKX*) genes, which degrade CK, while N scarcity reduces *IPT* expression and limits CK biosynthesis. Riboside-type CK, including zeatin riboside, is critical for long-distance transport under stress, ensuring CK delivery to sinks [83,84]. Serious drought-induced ABA accumulation antagonizes CK signaling by repressing CK-responsive genes such as *ARRs* (*Arabidopsis Response Regulators*). This antagonism shifts resource allocation from ear development to stress tolerance mechanisms, such as stomatal closure and soluble sugar accumulation. Nitrate transporters, such as *NRT1.1*, are modulated by CK, creating a feedback loop where CK enhances nitrate uptake under sufficient N but suppresses it under scarcity to conserve energy. Pre-treatment with CK analogs before drought or N stress could prime young ears, enhancing resilience without yield penalties [85,86]. Coupling split N application with deficit irrigation during the critical period could stabilize CK fluxes, which improve ear development compared to conventional methods in semi-arid regions. Critical knowledge gaps include the role of CK and its receptors in stress-specific signaling and the impact of soil microbiota on CK bioavailability. Emerging technologies, such as single-cell transcriptomics of ear meristems, could unravel spatiotemporal CK dynamics under combined stresses. Cytokinin acts as a double-edged sword in maize young ear development, balancing growth promotion and stress adaptation. By decoding their context-dependent mechanisms, future research will develop targeted strategies to optimize ear development under resource-limited conditions, ensuring sustainable maize productivity in a changing climate.

### 4.2. Cytokinin Mediates the Transmission of Nitrogen Signals from the Root System to the Shoot

**Figure 2:**
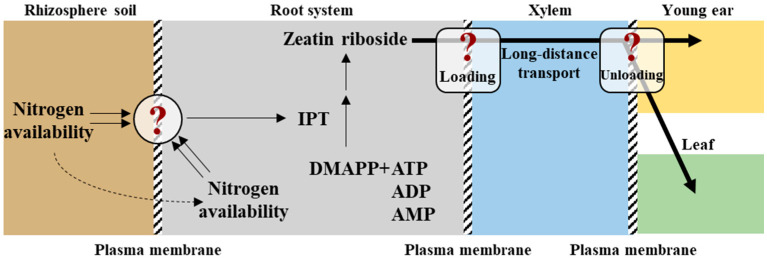
Model depicting the cytokinin-mediated transmission of nitrogen signals from the root system to the shoot. DMAPP: dimethylallyl pyrophosphate; ATP: adenosine triphosphate; ADP: adenosine diphosphate; AMP: adenosine monophosphate; IPT: isopentenyl transferase. The question in the circle represents the unclear mechanism of nitrogen perception and signal generation, and that in the rounded rectangle relates to regulatory mechanism of loading and unloading during the long-distance transport of cytokinin. The solid double arrows represent signal perception. The single solid arrows represent promoting effects or substance transformation. The dotted arrows represent positive correlation.

Plants have evolved sophisticated mechanisms to sense and respond to N fluctuations, with CKs serving as critical long-distance messengers that integrate root-derived N status with shoot developmental programs. CK is primarily synthesized in root tips, where N availability directly modulates its production. Key enzymes, such as *ISOPENTENYLTRANSFERASE* (*IPT*) and *LONELY GUY* (*LOG*), catalyze CK biosynthesis, with root-specific *IPT* expression upregulated under various N conditions [87]. In maize, for instance, nitrate sensing in roots triggers the synthesis of trans-zeatin (tZ), which is transported to shoots via the xylem to regulate leaf expansion and photosynthetic capacity. The spatial distribution of CK is tightly regulated by transporters such as *AtABCG14* in *Arabidopsis*, which facilitates the loading of iP ribosides, that is, CK precursors, into the xylem for systemic transport. In shoots, CK converts into tZ via LOG enzymes, activating downstream signaling cascades. This transport mechanism ensures that shoot tissues dynamically adjust their growth in response to root N status, optimizing resource allocation under fluctuating environments. CK regulates N-responsive genes through a phosphorelay system involving histidine kinases, such as *AHK3*; response regulators, such as *ARR1*; and transcription factors. In *Arabidopsis*, low-N conditions enhance CK degradation via *CYTOKININ OXIDASE/DEHYDROGENASE* (*CKX*), reducing shoot CK levels and repressing growth-promoting genes like *ARR12*. In contrast, nitrate application upregulates *IPT3* and *LOG4* in roots, increasing tZ that activate *ARR1*-mediated expression of *NRT2.1*. This feedback loop ensures efficient N acquisition and utilization under limited resource conditions. Notably, CK modulates shoot-to-root communication by regulating carbon allocation. Shoot-derived sugars enhance root *IPT* expression, promoting CK synthesis, which in turn stimulates nitrate uptake and assimilation. This C-N coupling underscores the role of CK in plant nutrient homeostasis. CK signaling intersects with auxin pathways to fine-tune N responses. CK antagonizes auxin-mediated lateral root growth by downregulating *PIN* auxin efflux carriers, redirecting resources to primary root elongation for deeper N foraging [88]. Conversely, N deficiency reduces CK levels, releasing auxin inhibition and promoting lateral root proliferation. Additionally, CK interacts with ABA to balance growth and stress responses. Under N limitation, ABA suppresses CK biosynthesis, prioritizing stress tolerance over shoot expansion [87]. Harnessing CK-mediated N signaling offers promising strategies to enhance NUE. The overexpression of *IPT* in roots under the control of nitrate-inducible promoters increases grain yield in rice by sustaining CK supply to shoots [89]. Similarly, CRISPR-edited *CKX* mutants in maize exhibit delayed senescence and improved N remobilization. Key challenges include elucidating the role of CK receptors in tissue-specific N signaling and deciphering how soil N availability influences CK bioavailability (Figure 2). Advances in single-cell transcriptomics and spatial metabolomics will unravel how CK gradients coordinate N responses at the cellular resolution.

### 4.3. Cytokinin Coupled with Nitrogen Promotes Grain Set in Maize

CK is pivotal in regulating maize ear development by stimulating cell division and differentiation in the apical meristem. During the early stages of ear development, CK promotes the proliferation of floret meristems, increasing the number of florets and enhancing ovule competence for fertilization [90]. Elevated CK levels during the critical period correlate with a 20–30% increase in fertile florets with exogenous CK application. This aligns with findings that CK upregulates *WUSCHEL*-related genes, which maintain cell pluripotency in meristematic tissues, thereby expanding the potential GN. N availability directly modulates CK synthesis and systemic signaling. In maize roots, nitrate sensing upregulates *IPT* genes, enhancing the production of tZ, which are transported via the xylem to developing ears. N deficiency suppresses *IPT* expression, reducing CK flux to shoots and impairing floret differentiation. This CK-N coupling is further evidenced by the role of *NRT1.1* in CK-mediated feedback loops [91,92]. During endosperm cellularization, CK coordinates with N to maximize the potential of GN. CK concentrations peak at the onset of endosperm cell proliferation, driving mitotic activity and increasing the number of endosperm cells, which determines the final grain weight [19,29,80]. Additionally, CK mitigates grain abortion during the late critical period. This is achieved through CK-mediated suppression of ethylene biosynthesis and reactive oxygen species accumulation, which is amplified by N availability. CK antagonizes auxin signaling in the ear apex, preventing excessive apical dominance and promoting uniform floret development. Simultaneously, CK coupled with brassinolide enhances vascular bundle differentiation, improving nutrient allocation to developing grains. CK upregulates sucrose synthase and invertase activities, ensuring carbon skeletons are available for amino acid synthesis [93,94]. In addition, CK enhances nitrate reductase activity, boosting nitric oxide production, which stabilizes auxin transporters and promotes floret survival. CK-responsive transcription factors (including *ARR1* and *ARR12*) integrate N status with developmental genes, such as *ZmEREB58*, thereby regulating endosperm cell division and grain set. By orchestrating meristem activity, endosperm development, and hormonal networks, CK-N coupling ensures a robust grain set even under suboptimal conditions.

### 4.4. Moderate Water Deficit May Synergistically Improve Water and Nitrogen Efficiency by Stimulating Grain Set Through Cytokinin

Water use efficiency (WUE) and nitrogen use efficiency (NUE) are critical determinants of agricultural productivity, particularly in water-limited and nutrient-constrained environments. In arid and semi-arid regions, drought conditions often enhance WUE by reducing evapotranspiration (ET) more significantly than net primary productivity (NPP), thereby promoting conservative water use. N assimilation is highly sensitive to water status, as hydraulic signals influence root uptake, translocation, and enzymatic activity. Under moderate water deficit, plants often exhibit increased N partitioning to grains, a response linked to CK signaling. Furthermore, irrigation experiments in China’s arid zones demonstrated that despite a 34.8% rise in irrigated water consumption over four decades, water productivity improvements mitigated trade-offs between yield and resource use [95]. Under moderate water deficit, root-derived CK is upregulated, promoting cell division in meristematic tissues and enhancing ovary viability, which promotes grain set. This hormonal adjustment compensates for reduced assimilate supply under water stress, ensuring reproductive resilience. Mechanistically, CK modulates aquaporin expression and stomatal conductance, optimizing water transport while sustaining photosynthetic activity. Additionally, CK interacts with ABA and other phytohormones to fine-tune stress responses. The synergy between WUE and NUE under moderate water deficit likely stems from CK-mediated coordination of carbon and nitrogen metabolism. CK enhances nitrate reductase activity and upregulates genes involved in nitrogen transport, ensuring efficient utilization even under limited water. Moreover, CK signaling promotes the expression of *ARR*-type response regulators, which integrate stress cues with developmental programs to prioritize young ear development over vegetative growth. A moderate water deficit emerges not as a yield-limiting constraint but as a physiological trigger to enhance resource efficiency through CK-mediated mechanisms. However, gaps persist in understanding spatial–temporal CK dynamics and their interaction with resource availability feedbacks.

## 5. Conclusions and Future Directions

**Figure 3:**
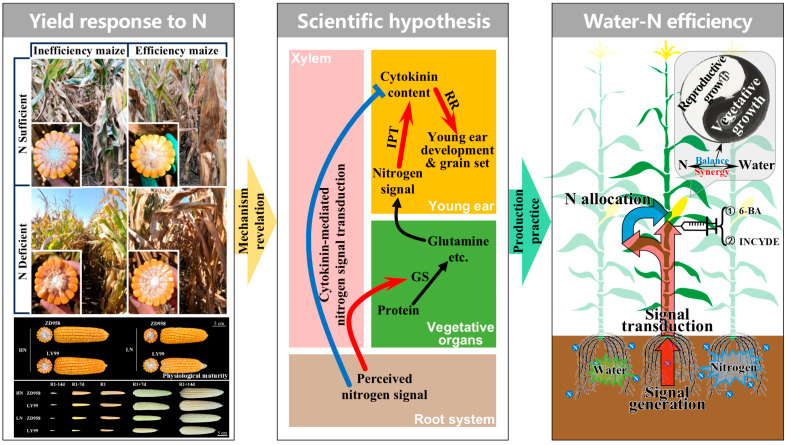
A schematic diagram illustrating cytokinin–nitrogen signaling networks governing ear differentiation, grain set, and yield potential in maize. GS: glutamine synthetase; IPT: isopentenyl transferase; RR: response regulator; 6-BA: 6-benzylaminopurine, that is, an artificially synthesized cytokinin-type plant growth regulator; INCYDE: a cytokinin dehydrogenase inhibitor. The red and blue arrows represent promotion and inhibition, respectively, while the black arrows represent substance transformation.

Maize production faces dual challenges of water scarcity and low nitrogen use efficiency, necessitating synergistic strategies to enhance resource utilization while ensuring yield stability. This review synthesizes the physiological mechanisms underlying water and N availability that regulate maize productivity, with a focus on the critical period of young ear development. Key findings highlight that GN determined during ear differentiation is the primary yield component sensitive to water and N stresses. Water deficits during this period impair photosynthetic carbon assimilation, disrupt hormonal homeostasis, and reduce the number of floret primordia, leading to significant GN losses. Similarly, N deficiency suppresses meristematic activity and floret differentiation. Importantly, the interaction between water and N management profoundly influences root architecture, phytohormonal dynamics, and metabolic reprogramming, underscoring the need for integrated approaches. Deficit irrigation emerges as a viable strategy to balance water conservation and yield stability. Moderate water deficit during the critical period enhances water use efficiency by optimizing stomatal conductance and root–shoot resource allocation. Notably, CK acts as a central hub linking water-N availability to ear development. This review also emphasizes the role of signaling over mere nutritional supply in regulating ear development and grain set. N availability during the critical period triggers CK and auxin-mediated pathways, modulating meristem activity and floret differentiation (Figure 3).

In order to collaboratively enhance the water and nitrogen use efficiency in maize production, future research should focus on elucidating spatiotemporal molecular networks. While hormonal and metabolic responses to water-N stress are partially characterized, the spatiotemporal coordination of these mechanisms remains unclear. Future studies should employ single-cell transcriptomics and spatial metabolomics to map hormonal gradients and redox states in young ear meristems during the critical period under dynamic stress conditions. Investigating epigenetic regulation, such as DNA methylation and histone modification, of stress memory could reveal priming strategies to enhance resilience. In addition, maize breeding programs should prioritize genes regulating CK biosynthesis (*IPT* and *LOG*), degradation (*CKX*), and signaling (*ARRs*), as well as redox regulators (*ZmGRX*). In production practice, sensor-based irrigation systems and machine learning models could predict crop water-N demand in real time, minimizing yield penalties. Combining moderate water deficit with N management may amplify WUE and NUE synergistically. Furthermore, rising temperatures and erratic rainfall necessitate research on drought-N interactions. For instance, elevated nighttime temperatures during the critical period accelerate developmental transitions but reduce floret fertility. Last but not least, scaling technologies like drip irrigation and/or efficiency-enhanced N fertilizers require addressing socioeconomic barriers, such as smallholder access to financing. Policy frameworks should incentivize climate-smart practices/cultivars through subsidies, farmer education, and infrastructure investment. On this basis, international collaborations should prioritize knowledge transfer and equitable resource governance.

In conclusion, achieving water-N synergy in maize production demands a multidisciplinary approach integrating physiology, genetics, agronomy, and socioeconomics. By unraveling the molecular basis of stress adaptation and deploying context-specific innovations, the global agricultural community can advance toward sustainable intensification, ensuring food security in a resource-constrained world.

## Data Availability

Not applicable.
